# Temperature-dependence on the optical properties of chitosan carbon dots in the solid state†

**DOI:** 10.1039/d0ra07779h

**Published:** 2021-01-13

**Authors:** Artur F. Sonsin, Sendy M. S. Nascimento, Itiara Mayra B. Albuquerque, Elaine C. O. Silva, José Carlos A. Rocha, Raissa S. Oliveira, Cintya D' Angeles E. S. Barbosa, Samuel T. Souza, Eduardo J. S. Fonseca

**Affiliations:** Optics and Nanoscopy Group, Institute of Physics, Federal University of Alagoas (UFAL) 57072-970 Maceió Alagoas Brazil arturfalqueto_sonsin@hotmail.com eduardo@fis.ufal.br; Institute of Chemistry and Biotechnology, Federal University of Alagoas (UFAL) 57072-970 Maceió Alagoas Brazil

## Abstract

We report the synthesis of chitosan-derived aminated carbon dots with dual fluorescence bands and their influence on the morphology, absorption and emission spectral profiles as well as on the band gap energy in relation to thermal treatment after synthesis. To unravel these changes, we performed spectroscopic measurements in the solid state on two stages at temperatures ranging from 303 to 453 K. For the first heating stage, the emission spectrum showed a 20 nm red shift and a new absorption band at 350 nm, possibly related to new bonds and/or nitrogenous molecular fractions. For the second heating stage in the same temperature range, no displacements in the emission spectrum were observed and both the energy gap and bandwidths for the two emission bands are practically constant, indicating a change nitrogen moiety exposed on the surface. Furthermore, through atomic force microscopy it was noted that the morphology and size of the CDs were not significantly affected by the increase in temperature. It is noteworthy that the values of the Huang–Rhys factor, respectively, 2.584 × 10^−10^ and 2.315 × 10^−9^ for band I and II emission after the second heating indicate a mechanism of weak electron–phonon interactions. This work may open a novel perspective for the development of new surface modulation strategies for carbon dots subjected to thermal treatment in the solid state.

## Abstract 2

Effect of thermal treatment on chitosan CDs in the solid state causes a redshift of PL emission and changes in surface states.

## Graphical abstract

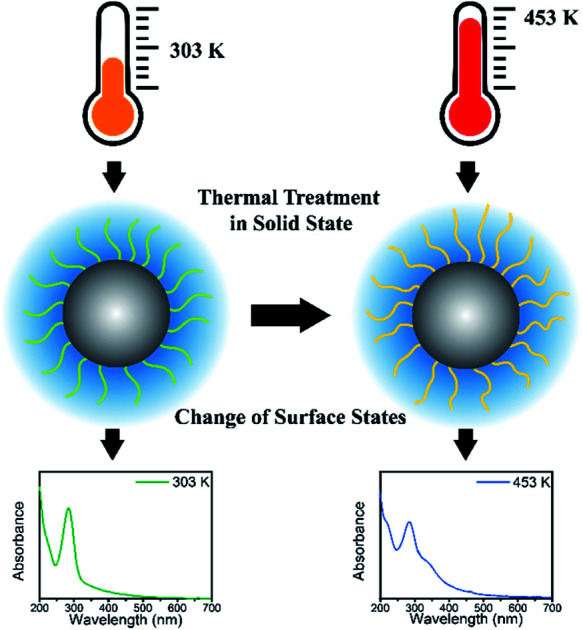

## Introduction

Carbon dots (CDs) are a class of carbon-based nanomaterials that have attracted researchers' attention because of their excellent optical and electronical properties, low cytotoxicity,^1^ excellent biocompatibility, and impressive photostability.^2^ Furthermore, the low cost, tunable excitation and emission wavelength, and superior solubility in water are some of the key features that have made research into them increase exponentially in the last 10 years.^3^

Due to their excellent photoluminescence (PL) properties, CDs have been shown to be immensely promising for diverse applications, particularly biological ones.^4–6^ They have been used as probes for different analytical uses^7^ and also in studies of photocatalysis, energy conversion, optoelectronics, and sensing.^6,8–11^ Scientific papers about new applications and reviews of CDs have greatly increased since their discovery.^12–14^ CDs have also several advantages over the already well-known quantum dots (QDs), such as neglectable cytotoxicity as well as easy and inexpensive synthesis. These properties make them strong competitors to QDs, because they can be used as a new type of fluorescent material to replace QDs in many applications.^12,15^

The optical properties of CDs have been extensively investigated and most of works has been focused on PL associated with the variety of synthesis procedures.^16,17^ CDs emission spectrum may or may not be dependent of the excitation wavelength and the surface structure is considered to be the main reason for that. Some studies have shown a ratio between PL intensity and particle size,^2,18–22^ surface states^1–3,23–25^ and heteroatom (presence of oxygen, nitrogen or sulfur).^1,2,26,27^ These properties can be defined during the synthesis process or through some type of treatment after synthesis.

The PL spectrum of CDs show excitation-, pH-, solvent- and temperature dependence.^3,18,19,26,28^ It is important to notice that is possible to do some kind of treatment after CDs synthesis such as chemical reaction, different solvent, add ions, pH values or temperature.^3,18^ Among such effects, the temperature is very prospected to understand the CDs' photophysical properties and for application in the field of thermometry. In the most common cases the temperature leads to a decrease in the PL intensity and/or changes in the spectral profile of CDs, such as the shift of the emission band.^2,3,19^

Regarding PL decreasing in function of temperature, the most clear explanation is the occurrence of thermally activated nonradioactive relaxation.^2,3^ Some works showed that the shift of emission spectra is a relationship between CDs growth and temperature increases. Wang *et al.* synthesized stable CDs by hydrothermal treatment of glucose (glc) in the presence of glutathione (GSH) and noted a reversible red shift emission upon temperature cycling in the biological range (288–363 K).^19^ Additionally, different content, ratios or speciation of heteroatoms in the surface or core of CDs can be changed when the temperature is varied either before or after the CDs formation.^29^ In addition, it is worth noting that studies related to changes in optical and morphological properties of CDs in the solid state with temperature variation are rare. One of these studies was developed by Yu *et al.*^3^ By varying the temperature from 77 to 300 K, they showed that, with increasing the temperature, the intensity of the PL decreases, thus causing a small red shift.

Nowadays the use of organic chemicals, the so-called green chemistry concept have been widely desired.^30^ These syntheses generally use cheap and renewable resources as raw material, such as orange juice, cow manure, chicken eggs, *etc.*^31–35^ A raw material very used in the synthesis of CDs is chitosan. Chitosan is a biopolymer derivative of chitin, an organic compound abundant in nature and can be extracted from the exoskeleton of some crustaceans and insects. This material contains high amounts of amino (–NH_2_) and hydroxyl (–OH) functional groups. Therefore, the synthesis of the CDs from this compound has an amount of nitrogen (amine, pyridinic nitrogen or pyrrolic nitrogen).^36^ These functional groups containing nitrogen further improve the luminescent properties, which make chitosan an interesting raw material in the synthesis of these nanostructures.^26,36^

In the present work, the amino-functionalized-CDs with dual fluorescence bands and excitation-dependent emission have been synthesized *via* hydrothermal method from chitosan. Here our study aims to understand the effect of thermal treatment on PL emission, absorption spectrum, band gap energy and size of CDs in the solid state. The researches in this field are still a challenge due to the complex emission mechanism as well as by the compositional and structural diversification of CDs. Thus, our findings can contribute considerably in a very scarce research field.

## Experimental

### Synthesis of carbon dots

Carbon dots were synthetized *via* hydrothermal method by using chitosan gel as precursor material. Firstly, 0.5 g of chitosan (Sigma-Aldrich) was dissolved under magnetic stirring for 30 min in 100 mL of a glacial acetic acid solution (10%) (Sigma-Aldrich ≥99.7%), resulting in a pale-yellow colored gel. A volume of 12 mL of this gel was transferred into 25 mL teflon-lined stainless steel autoclave and heated at 453 K for 6 h. When the reaction was finished, the dark brown solution containing the CDs was naturally cooled, centrifuged at high speed (15.000 rpm) for 15 min and filtered through the 0.22 μm porosity filter. The obtained CDs were stored at 277 K.

### Samples preparation to thermal treatment after synthesis

CDs supported on silicon and MICA substrates were prepared to perform the measurements of the temperature-dependent PL spectra, transmission electron micrograph images and AFM images. In the preparation process of CDs film, 25 μL of CDs solution was deposited drop by drop in a silicon and MICA substrates. After 15 min in the desiccator, a CDs film was formed.

In the preparation process of CDs suspension, 200 μL of CDs solution was added in Petri dish and heated in a muffle with appropriate temperature. 30 min after achieving the temperature of 303 and 453 K, CDs were removed in solid-state. Subsequently, 2 mL of distilled water was used to solubilize these CDs. The CDs exposed to this process were used in the measurements of the excitation-dependent PL spectra, UV-vis absorption spectra and FTIR spectra.

### Characterization of carbon dots

#### Photoluminescence

For the measurements of the excitation-dependent fluorescence spectra, CDs suspension were measured using a FluoroLog®-3 spectrofluorometer (HORIBA, Kyoto, Japan) equipped with a xenon lamp (CW 450 W), a detector photomultiplier (model R928P) and using an excitation wavelength ranging from 310 to 532 nm. The CDs suspension was externally heated at 303 and 453 K. After solubilize, the fluorescence was measured for both cases at room temperature.

For the measurements of the temperature-dependent fluorescence spectra, CDs film were recorded using a Xplora (HORIBA, Kyoto, Japan) equipped with a laser of 532 nm, lens 10× objective (NA = 0.3 and WD = 17.5 mm). A heater (INSTEC hot and cold stage) was coupled to the Xplora. The spectra were collected during sample heating from 303 K to 453 K, varying of 30k. After the first cycle heating, the sample was cooled to room temperature.

#### Fourier transform infrared spectroscopy (FTIR)

The infrared spectra of the CDs suspension were obtained using an IRPrestige-21 spectrophotometer (Shimadzu, Kyoto, Japan) coupled with an attenuated total reflectance (ATR) accessory with ZnSe crystal. The spectral range was 4000–800 cm^−1^, 4 cm^−1^ resolution and 120 scans. The bands intensities were expressed in transmittance (%) with a diffuse reflectance accessory (DRS-8000).

#### UV-vis spectroscopy

Absorption spectra of the CDs suspension were performed by an UV-3600 spectrophotometer (Shimadzu, Kyoto, Japan). The spectra were recorded in the range of 200–700 nm.

#### Atomic force microscopy (AFM)

AFM images of the CDs were obtained in a pattern configuration of the Multiview 4000™ atomic force microscope (Nanonics, Israel) with a combined optical microscope (BXFM, Olympus, Japan). The AFM system was acoustically isolated to reduce some interference during the measures caused by the noise of the environment. The instrument was fixed in an active damping table to suppress mechanical noise. The topography (256 × 256 pixels) of the sample was analyzed in the tapping mode with a 0.3–1 Hz scan rate. AFM images were processed with WSxM software.^37^

#### Transmission electron microscopy (TEM)

Transmission electron micrograph images were taken using a JEM-2100 transmission electron microscope (JEOL, Tokyo, Japan) operating at an accelerating voltage of 200 kV. The solutions of the CDs were dropped on a 400-mesh C-coated copper grid (Ted Pella Inc., Redding, CA, USA) and dry under ambient conditions.

#### Energy dispersive X-ray (EDX)

Scanning electron microscope SUPERSCAN SSX-550, with integrated EDX and anti-vibration workstation (Shimadzu). The sample was deposited on a silicon wafer so that there is less interference with the surface.

### Quantum yield measurement

Diluted CDs solutions were employed for calculation of QY, using quinine sulfate as standard. The absorbance was kept less than 0.1 (to avoid deviation line of absorbance and the molar concentration), with excitation wavelength of 320 nm. CDs were placed in a 10 mm cuvette in order to minimize the resorption effects. Then, the integrated photoluminescence intensity was calculated as the area under the PL curve in the wavelength range from 340 to 600 nm. The results were plotted with the integrated photoluminescence intensity *vs.* corresponding absorbance to obtain the slope of the curve for the CDs and the standard (Fig. S1†). The slopes of the linear plots were used to calculate QY using the following expression:^38–40^1
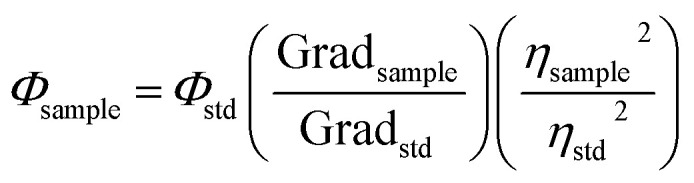
where std index denotes the standard (quinine sulfate) and sample the CDs. *Φ* is the QY, Grad is the gradient from the linear regression analysis, and *η* is the refractive index. The refractive index of solvent used for CDs (water) is equal to 1.33. QY of quinine sulfate was taken as 0.54 and the refractive index of solution, prepared in 0.1 M H_2_SO_4_, was taken as 1.33.

## Results

### Characterization of carbon dots

**Fig. 1:**
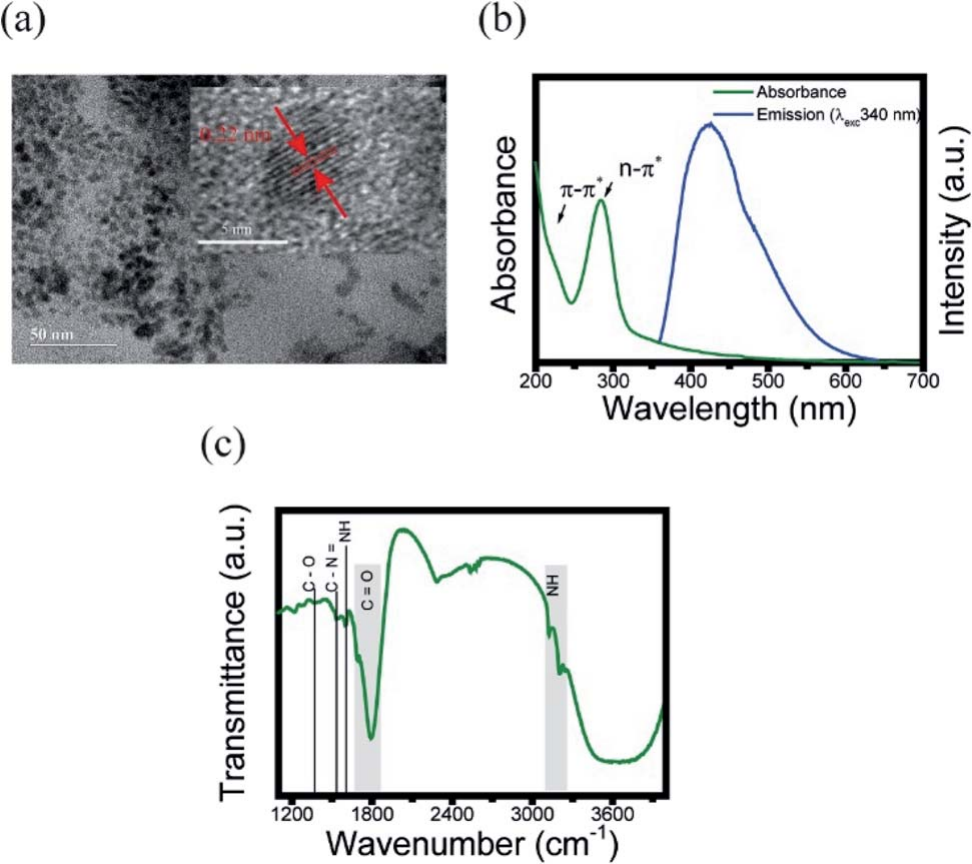
Characterization of CDs. (a) TEM images of CDs. Inset: lattice spacing; (b) UV-visible absorption (green line) and emission (blue line) spectra of CDs; (c) FTIR spectrum at 303 K.

In order to obtain the information of physical and chemical properties of CDs, we performed some methods of characterization. Herein, TEM imaging, UV-vis and FTIR spectroscopies were used to evaluate the morphological and optical properties of CDs, respectively. The presence of CDs was confirmed with TEM, as shown in Fig. 1a. This technique was used to analyze the size, morphology and graphitic structure of the synthesized CDs from the carbonization of chitosan. Their size was around 6 nm with quasi-spherical shaped particle. The lattice spacing is 0.22 nm similar to the interlayer distance in graphite (the inset of Fig. 1a),^41^ such as the majority CDs.

To support the characterization of the CDs, UV-visible spectroscopy was used in order to identify the characteristic absorptions of the core and surface of the CDs. The absorption spectrum (Fig. 1b) demonstrate a characteristic peak in 285 nm, corroborating the range common between 250–300 nm, that is known as a n–π* transition peak.^3,26^ Meanwhile, the peak in 221 nm corresponds to π–π* in the surface region, which may originates from the starting material (chitosan).^19,26^

Next, the chemical composition and functional groups present on the surface of the CDs were identified using FTIR. Fig. 1c shows the FTIR spectra of the CDs suspended at 303 K. These spectra show a strong absorption band at around 1790–1670 cm^−1^ assigned as the stretching vibrations of C=O from amide, the broad band at 3600 cm^−1^ originated from the OH bond, and the band between 3200–3100 cm^−1^ corresponds to the NH originating from secondary amines. The band at 1604 cm^−1^ is assigned to aromatic groups C=C, 1530 cm^−1^ corresponds to the NH bond, 1363 cm^−1^ band is assigned to the C–N= bond, and the 1221 cm^−1^ band originates from the C–O bond.^26^ The band between 2500–2600 cm^−1^ corresponds to the CO_2_ present in the atmosphere.^42^ Additionally, the value of absolute quantum yield (QY) was measured to be 4.36%, which was comparable to the reported CDs.^26,43,44^ EDX and corresponding elemental mapping analysis were further used to confirm the presence of the elements C, O and N (Fig. S2†).

### Photoluminescence (temperature-dependent) of carbon dots

**Fig. 2:**
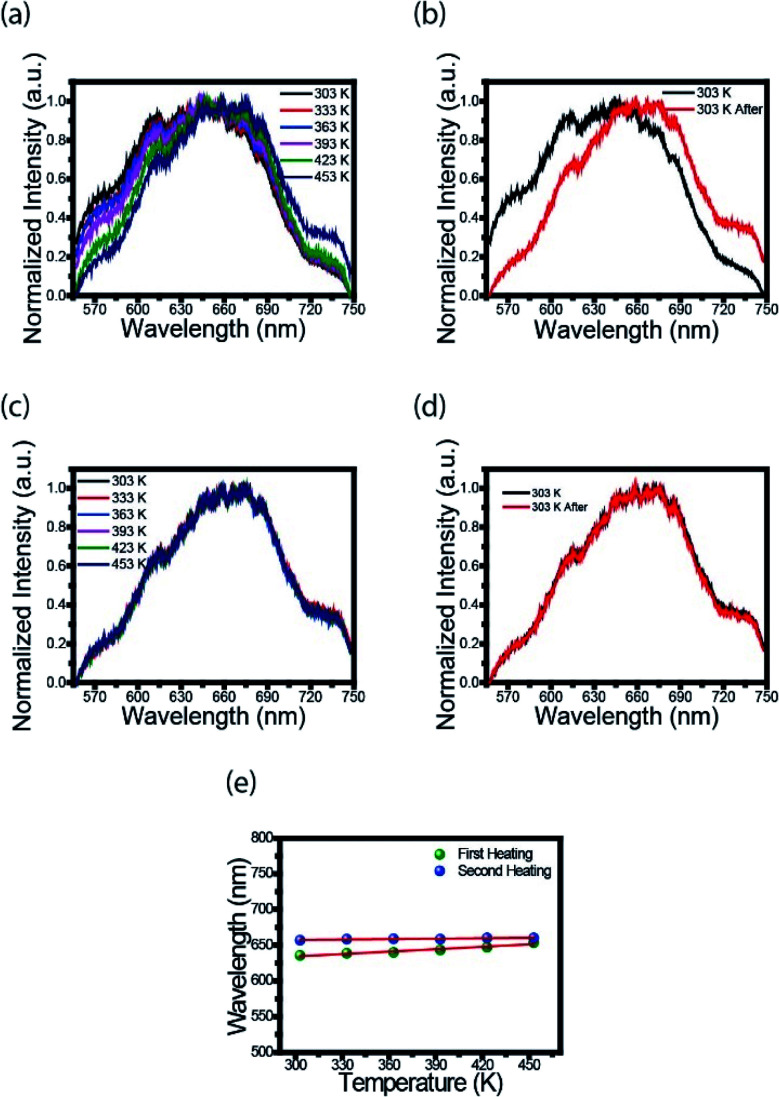
PL with 532 nm excitation varying temperature. (a) First heating; (b) before and after first heating; (c) second heating; (d) before and after second heating. (e) The emission wavelength of spectra higher intensity for each temperature.

To further explore the optical properties of the CDs, photoluminescence (PL) spectra were studied. Fig. 2a shows the fluorescence spectra of CDs film measured with Xplora spectrometer using a green (532 nm) light laser source. These measurements were taken at the temperatures described in the legend using a heater coupled to spectrometer. It is clearly observed a 20 nm red shift, from the black to the dark blue line, when the temperature is increased. This effect can be attributed to the surface changes. It is possible that the first heating may have modified the surface, reducing the defects and/or creating new states. The black line profile (Fig. 2b) is the fluorescence spectrum at 303 K and the red line corresponds to the spectrum of CDs film heated to 453 K, cooled down and measured at 303 K. This is an important point because for the first heating stage the process is irreversible.

Therefore, once the sample is heated after synthesis, the luminescence emission of the CDs changes to a longer wavelength, and, after cooling, the PL profile does not return to the spectrum before heating. This photoluminescence spectra change is permanent, even for the biological range in contrast to the results obtained by Wang *et al.*^19^

Fig. 2c shows the spectrum of CDs films in the second heating stage. The PL profiles are the same after the second heating; they keep the same for different temperatures. This result is evident in Fig. 2d, *i.e.*, there is no red shift effect. The heating acts like a thermal treatment that permanently changes the luminescence of CDs. Fig. 2e shows the variation of the highest intensity peak in the spectrum as a function of temperature. In the first heating (green dots) is observed an increase in wavelength from 635 to 655 nm. For the second heating (blue dots) no red shift is observed, as previously described.

The expression proposed by O'Donnell and Chen is used to describe the temperature dependence of the band gap in semiconductor, which is based on the analysis of electron–phonon coupling mechanism responsible for the band gap shift.^45^ Yu *et al.*^3^ studied temperature-dependent fluorescence, on excitation-independent CDs, between 77 and 300 K. A small Huang–Rhys factor was observed, implying in a weak exciton–phonon coupling. Furthermore, the temperature-independent bandwidth suggested a strong electron–electron interaction.

**Fig. 3:**
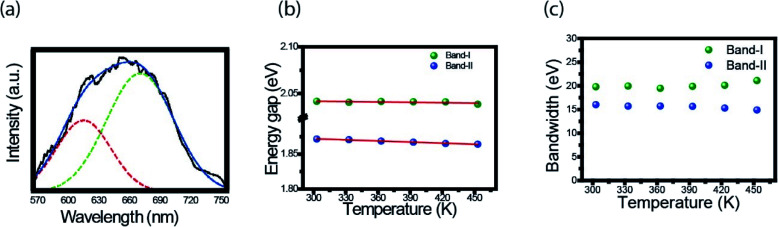
(a) Emission spectrum (303 K) fitted by a two-Gaussian functions. The band I is attributed to the core emission and band II to the surface state emission; (b) energy gap and (c) bandwidth, both as a function of temperature in CDs.

Here we follow the idea presented by Yu *et al.*^3^ It is possible to evaluate the contribution of the nucleus and of the surface states from the fluorescence spectrum. The asymmetric peaks are clearly observed in Fig. 2a and each emission spectrum can be well fitted by two-Gaussian functions, as shown in Fig. 3a for the 303 K spectrum. The band I is attributed to the core emission and band II to the surface state emission. We performed the same procedure for the other spectra form Fig. 3a, in such way that the peaks of the bands (in energy unity) can be plotted as function of temperature, as shown in Fig. 3b. The two bands show temperature dependence, evidencing the red shift effect. From these points, we used the following equation,^46^2
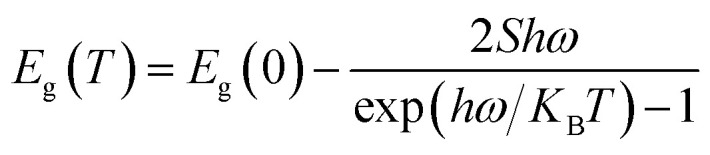
where *S* is the Huang–Rhys factor that represents the exciton–phonon coupling strength, *hω* is the average phonon energy, and *K*_B_ is the Boltzmann constant. By fitting the points of each band with this equation, we obtain the following fit parameters: *E*_g_ (0) = 2.044 eV, *hω* = 21.4 meV, and *S* = 2.584 × 10^−10^ for band-I and *E*_g_ (0) = 1.888 eV, *hω* = 10 meV, and *S* = 2.315 × 10^−9^ for band-II. The small value of *S* represents a weaker electron–phonon coupling in CDs, which is consistent with our observation of a small red shift, in contrast with QDs with bigger red shift effect. For example, InP/ZnS and CdSe/CdS, with *S* value of 1.95 and 1.57, respectively.^46–48^

Another important parameter that can be extracted from the bands I and II of Fig. 3a are their bandwidths. Fig. 3c presents the band-I and band-II bandwidths as a function of temperature, showing that this parameter does not exhibit a significant variation in temperature. Previous studies in semiconductor and QDs showed that the bandwidth of the spectra can be separated into homogeneous and inhomogeneous terms.^49,50^ Homogeneous broadening depends on temperature and it is related to the dispersion of excitons by acoustic and optical phonons. Inhomogeneous broadening, on the other hand, stems from fluctuations in the size, shape and composition of the QDs. This is ascribed on bandwidth profile as temperature independence which indicates that electron–electron scattering dominates. The total bandwidth can be described using the following expression.^3,46,47,49,50^3
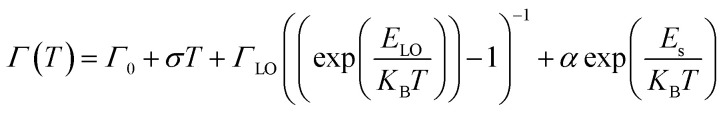
where *Γ*_0_ (electron–electron scattering) is the temperature-independent intrinsic term (inhomogeneous) and the last three terms represent temperature dependent terms (homogeneous), *σ* is the electron–acoustic phonon coupling coefficient, *Γ*_LO_ represents the strength of exciton–LO phonon coupling, *E*_LO_ is the LO phonon energy, *α* is the bandwidth due to fully ionized impurity/defect scattering, and *E*_s_ is the activation energy for ionization. As seen in Fig. 3c, the bandwidth of bands I and II has a small variation, as a consequence the bandwidth did not considerable variation in temperature. This result indicates that electron–electron scattering dominates and the phonon coupling is very small, as previously mentioned on the energy gap analysis.

### Photoluminescence (excitation-dependent) of carbon dots

**Fig. 4:**
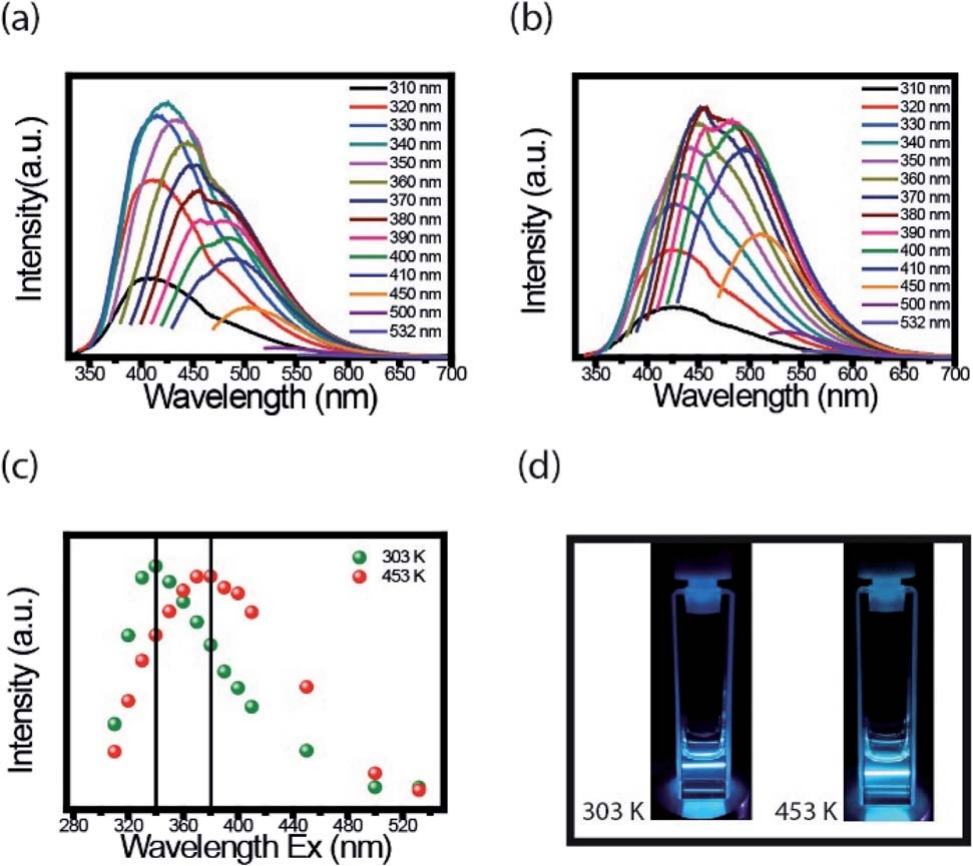
Excitation-dependent PL emission spectra. (a) Heating 303 K and (b) heating 453 K. (c) The wavelength of highest intensity from emission-dependent PL at 303 K, plot (a), (green dots) and 453 K, plot (b), (red dots). (d) The photographs of fluorescent CDs under laser excitation 380 nm.

An interesting property in CDs is to investigate the PL spectra for different excitation wavelength. We performed the measurements at room temperature with the FluoroLog®-3 spectrofluorometer. For these measurements' CDs solution was firstly externally heated (303 K and 453 K) and after resuspended in water. Fig. 4a and b present the PL emission spectra of CDs suspension heated to 303 K and 453 K, respectively. Based on the results, the dependence of each spectrum with the excitation wavelength is evident.

Fig. 4c presents the maximum intensity of each spectrum, shown in Fig. 4a and b, as a function of the excitation wavelength. The CDs suspensions, the ones heated to 453 K and cooled to room temperature, had the spectra shifted to a longer wavelength compared to spectra of CDs heated at 303 K. The highest value intensity changed from 340 to 380 nm after heating. Both CDs suspension have blue fluorescence under 380 nm UV light illumination, but for heated CDs, this fluorescence is a little brighter (Fig. 4d). Both images were taken at room temperature. The important point here is, even though, the red shift maybe has different mechanisms in the solid and liquid states, in both cases this effect was observed.

### AFM of carbon dots

**Fig. 5:**
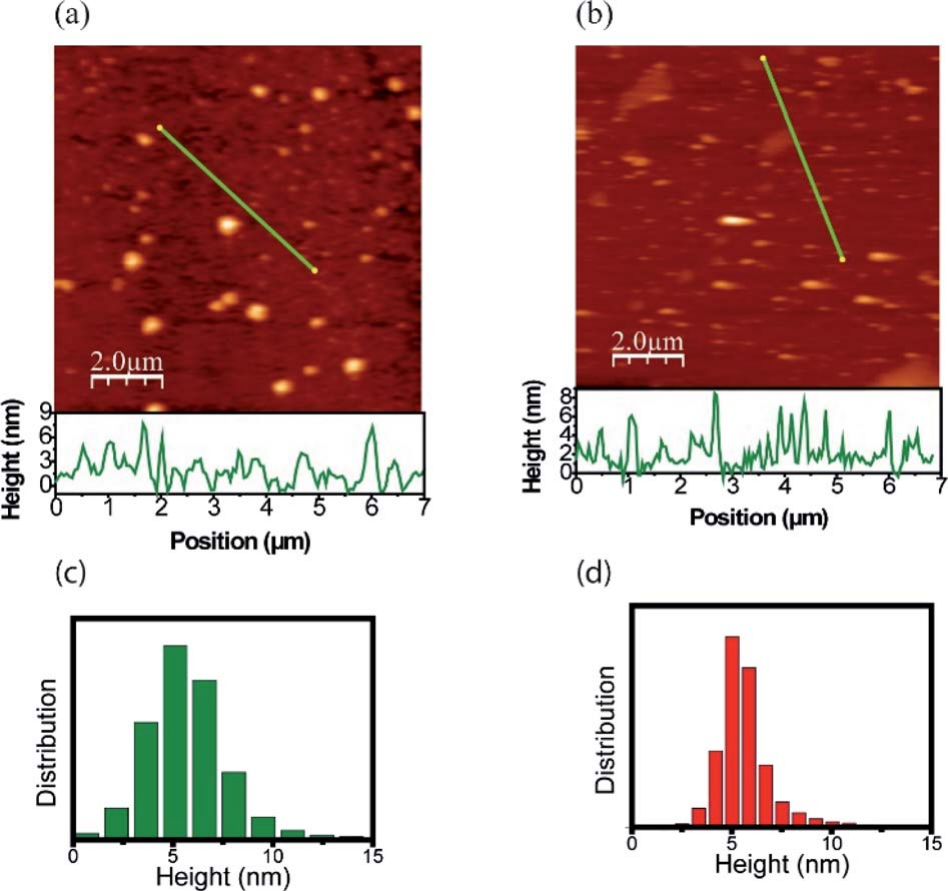
AFM images of CDs at different temperatures: (a) 303 K and (b) 453 K. The measured height profile along the green line is also shown. Height distribution histograms of CDs: (c) 303 K and (d) 453 K.

**Fig. 6:**
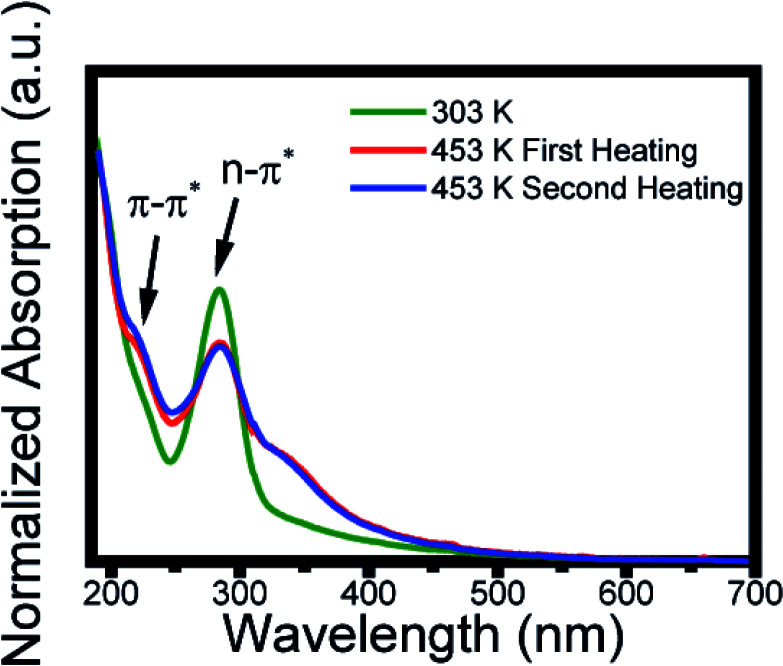
Absorption spectra at 303 K (green line), after the first heating at 453 K (red line), and the second heating at 453 K (blue line).

The morphology and size distribution of the CDs were also analyzed by AFM. Fig. 5 shows AFM images of the CDs after being heated to 303 K and 453 K. Fig. 5a shows the CDs at a temperature of 303 K, where the brightest points represent clusters. To better analyze its size, the profile is drawn in a region without clusters, where the CDs are more dispersed. From height distribution histogram in Fig. 5c, it is possible to observe that the average height of the CDs is approximately 6 nm. Fig. 5b shows the AFM image of the CDs after being heated to 453 K. Its height distribution histogram is shown in Fig. 5d, where the average height obtained is approximately 6 nm. Therefore, the size remains unchanged after heat treatment. The AFM images confirm that the quantum confinement (size) was not responsible for the changes in the PL. Thus, the inhomogeneous component is not due to the change in size and shape and it can be inferred that PL has a greater contribution from the surface. This result is in accordance with the data obtained in the UV-vis analysis after the thermal treatment of the CDs and can be visualized in Fig. 6.

### UV-vis spectrum of carbon dots

The carbon dots solution as prepared at 303 K (green line) showed two typical absorption bands located at 221 and 285 nm, assigned, respectively, to the π–π* transition of aromatic sp^2^ domains from the carbon core and to the n–π* of the surface moieties.^26^ After the first heating to 453 K (red line) the spectrum profile presents a change in the absorption profile displaying a new band at 350 nm. This absorption remains after the second thermal treatment, which indicates a change in its surface state.^51–54^ Thus, the red shift (Fig. 2a) and the change in the peak of greater intensity (Fig. 3c) caused by the increase in temperature are due to the change in surface states. Studies have shown that the absorption band at 350 nm is caused by a nitrogen-rich environment on the surface.^54–56^ This is an indication that changes in surface states are due to the amine groups. The inhomogeneous bandwidth shown in Fig. 3c, also corroborates the changes in the surface state, since it is related to fluctuation in the composition. To reinforce the conclusion about the surface modification, it was performed FTIR spectrum after heating and the data obtained do not show significant variation (Fig. S3†). EDX and corresponding elemental mapping analysis after heating showed the presence of C, O and N, proving that there is no elementary change in the CDs (Fig. S4†).

## Conclusion

In this study, we report changes in photoluminescent properties of CDs in the solid state with thermal treatment. We found that with the increase in temperature, its PL emission has a permanent red shift. The low Huang factor means a weak electron-phonon coupling, revealing that quantum confinement is not the main mechanism of PL emission, fact which was confirmed by the AFM images when observed that CDs size do not change. The non-dependence of the bandwidth of CDs in the solid state with the thermal treatment showed that the electron–electron dispersion dominates, therefore its PL is indicated by changes in its surface state, which was also confirmed through the absorption measures. Finally, this work opens new possibilities for photophysical alterations of CDs using an unusual approach in the literature of thermal treatment in solid state. Furthermore, this strategy could be explored for optical applications in the future.

## Author contributions

Artur F. Sonsin was responsible for the measurements, data analyses, writing, review and editing the manuscript. Sendy M. S. Nascimento helped in data analysis and writing. Itiara Mayra B. Albuquerque helped in the analysis of FTIR data and writing. Elaine C. O. Silva was responsible for the writing, review and editing the manuscript. José Carlos A. Rocha was responsible for the computational assistance. Raissa S. Oliveira was responsible for the carbon dots synthesis. Cintya D' Angeles E. S. Barbosa helped in conceptualization, writing, review and editing the manuscript. Samuel T. Souza helped with AFM topography and image acquisition, as well as data supervision. Eduardo J. S. Fonseca was the responsible for project administration, writing the original draft and general supervision. All authors read and approved the final manuscript.

## Conflicts of interest

There are no conflicts to declare.

## Supplementary Material

RA-011-D0RA07779H-s001
