# Supplementary material for: Temperature-dependence on the optical properties of chitosan carbon dots in the solid state
Source: RSC Adv. 2021 Jan 13;11(5):2767–73. doi: 10.1039/d0ra07779h (PMC8693838; doi:10.1039/d0ra07779h)
Supplement: RA-011-D0RA07779H-s001 [file RA-011-D0RA07779H-s001.pdf]

Electronic Supplementary Material (ESI) for RSC Advances.
This journal is © The Royal Society of Chemistry 2021

Temperature - Dependence on the optical properties of chitosan carbon dots in the solid state

Artur F. Sonsin[a]*, Sendy M. S. Nascimento[a], Itiara Mayra B. Albuquerque[a], Elaine C. O. Silva[a], José Carlos A. Rocha[a], Raissa S. Oliveira[b], Cintya D' Angeles E. S. Barbosa[b], Samuel T. Souza[a], Eduardo J. S. Fonseca[a]*

[a]Optics and Nanoscopy Group, Institute of Physics, Federal University of Alagoas (UFAL), 57072-970, Maceió, Alagoas, Brazil

[b]Institute of Chemistry and Biotechnology, Federal University of Alagoas (UFAL), 57072-970, Maceió, Alagoas, Brazil.

*E-mail: arturfalqueto_sonsin@hotmail.com; and eduardo@fis.ufal.br

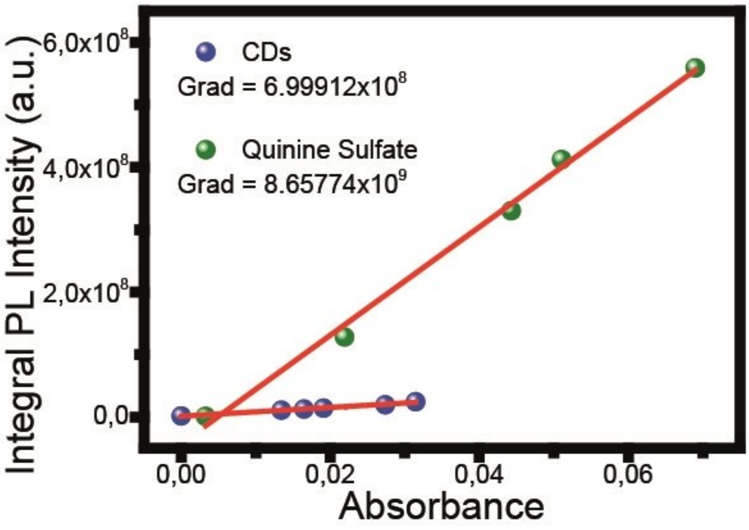

Figure S1: Plot of fluorescence intensities against their absorbances for CD and standard quinine sulfate.

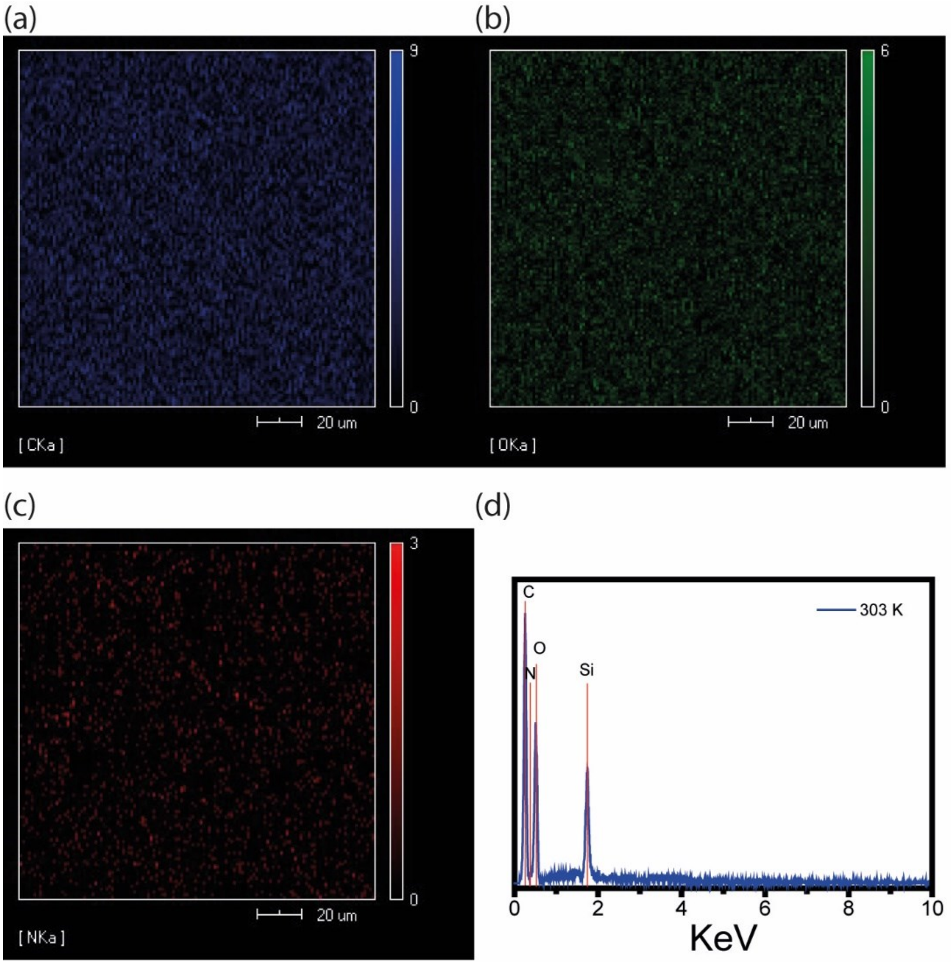

Figure S2: Elementary mapping of CDs corresponding to (a) carbon, (b) oxygen, and (c) nitrogen. (d) EDX spectrum of CDs at 303 K.

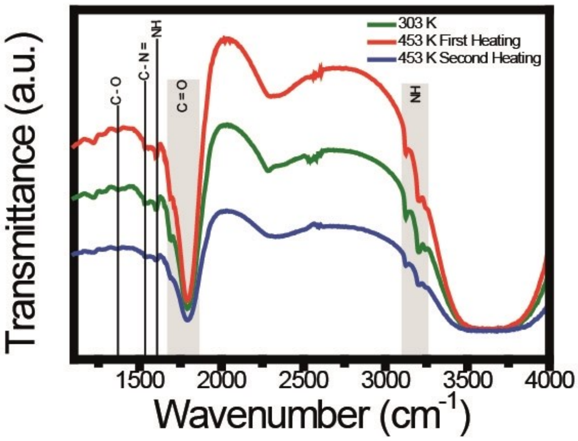

Figure S3: FTIR spectrum at 303 K (green line), after the first heating at 453 K (red line), and the second heating at 453 K (blue line).

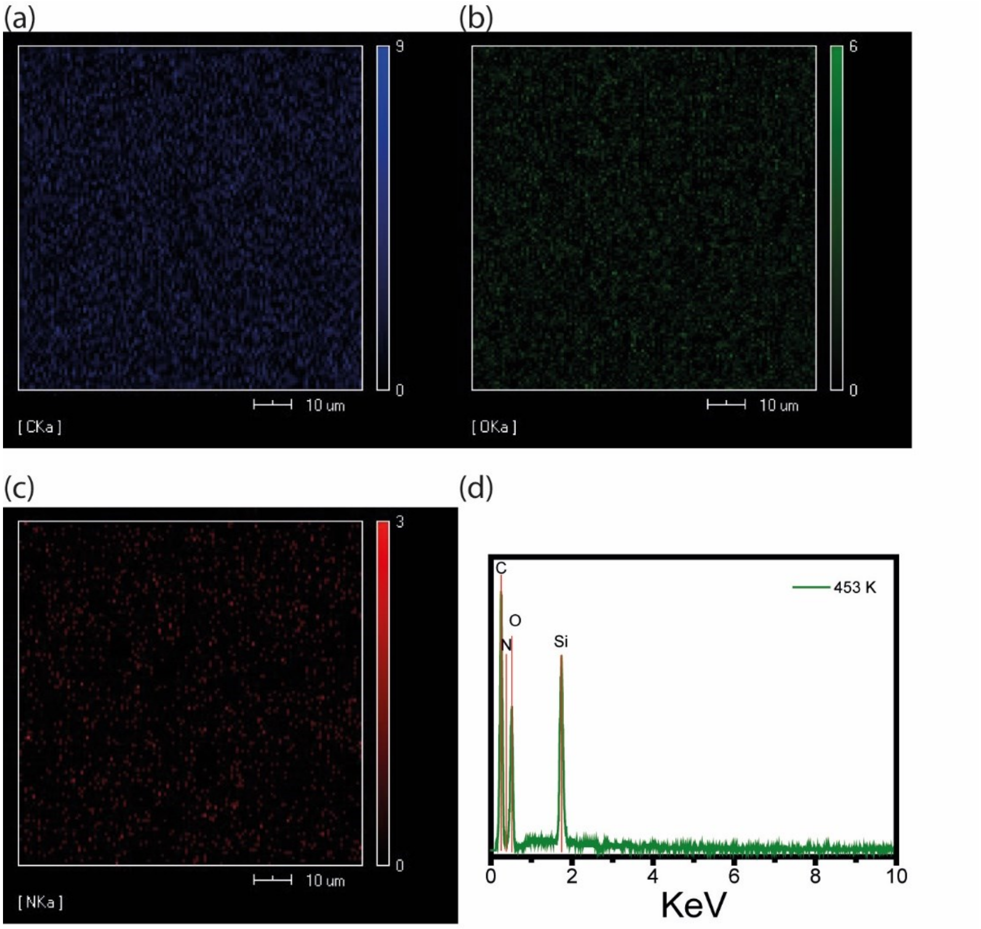

Figure S4: Elementary mapping of CDs corresponding to (a) carbon, (b) oxygen, and (c) nitrogen. (d) EDX spectrum of CDs at 453 K.
